# A Probiotic Amylase Blend Positively Impacts Gut Microbiota Modulation in a Randomized, Placebo-Controlled, Double-Blind Study

**DOI:** 10.3390/life14070824

**Published:** 2024-06-28

**Authors:** Mahmoud A. Ghannoum, Mohammed Elshaer, Hilmi Al-Shakhshir, Mauricio Retuerto, Thomas S. McCormick

**Affiliations:** 1 Department of Dermatology, School of Medicine, Case Western Reserve University, Cleveland, OH 44106, USA; 2 Department of Dermatology, University Hospitals Cleveland Medical Center, Cleveland, OH 44106, USA; 3 Department of Clinical Pathology, Faculty of Medicine, Mansoura University, Mansoura 35516, Egypt

**Keywords:** gut health, bacteria, fungi, microorganisms, *Candida*

## Abstract

The present study was performed to determine if ingesting a blend of probiotics plus amylase would alter the abundance and diversity of gut microbiota in subjects consuming the blend over a 6-week period. 16S and ITS ribosomal RNA (rRNA) sequencing was performed on fecal samples provided by subjects who participated in a clinical study where they consumed either a probiotic amylase blend (*Bifidobacterium breve* 19bx, *Lactobacillus acidophilus* 16axg, *Lacticaseibacillus rhamnosus* 18fx, and *Saccharomyces boulardii* 16mxg, alpha amylase (500 SKB (Alpha-amylase-Dextrinizing Units)) or a placebo consisting of rice oligodextrin. The abundance and diversity of both bacterial and fungal organisms was assessed at baseline and following 6 weeks of probiotic amylase blend or placebo consumption. In the subjects consuming the probiotic blend, the abundance of *Saccharomyces cerevisiae* increased 200-fold, and its prevalence increased (~20% to ~60%) (*p* ≤ 0.05), whereas the potential pathogens *Bacillus thuringiensis* and *Macrococcus caseolyticus* decreased more than 150- and 175-fold, respectively, after probiotic-amylase blend consumption. We also evaluated the correlation between change in microbiota and clinical features reported following probiotic amylase consumption. Nine (9) species (seven bacterial and two fungal) were significantly (negatively or positively) associated with the change in 32 clinical features that were originally evaluated in the clinical study. Oral supplementation with the probiotic-amylase blend caused a marked increase in abundance of the beneficial yeast *S. cerevisiae* and concomitant modulation of gut-dwelling commensal bacterial organisms, providing the proof of concept that a beneficial commensal organism can re-align the gut microbiota.

## 1. Introduction

Microbial specificity depends upon habitat, and this is also true for commensal organisms that colonize the intestinal tract (gut). The gut represents a rich niche for microorganisms, where polymicrobial interactions between bacterial (bacteriome) and fungal (mycobiome) communities occur [1]. Given these polymicrobial interactions, the gut represents quite a diverse host locale, which facilitates microbial and fungal interactions. However, diversity represented within the gut can be altered by intrinsic and extrinsic factors, including diet, lifestyle, overall health, exogenous therapeutics, or other environmental factors.

Probiotics, live microorganisms that reside primarily in the gastrointestinal tract and support host health when taken in adequate amounts, are becoming a popular dietary supplement [2]. Probiotics have been used for over a century [3] and are found in supplement form as well as in foods. 

Probiotics modulate the gut microbiota in several potentially beneficial manners, such as increasing the abundance of beneficial bacteria and yeast, fortifying the mucus layer to improve the physiological barrier function, keeping pathogens in check, and stimulating the immune response [4]. Probiotics have also been associated with beneficial effects on anxiety, stress, and mood via signaling associated with the gut–brain axis [5]. Microbial benefits are often associated with specific strains of organisms, and therefore testing strain-specific effects and formulating a mixture of strains with/without other ingredients (e.g., enzymes, prebiotics) to provide an optimal benefit for the host at an effective dose is necessary [6].

Recently, it was demonstrated that consumption of a novel formulation of probiotic strains (*Bifidobacterium breve* 19bx (1.3 billion colony forming units (cfu), *Lactobacillus acidophilus* 16axg (12.8 billion cfu), *Lacticaseibacillus rhamnosus* 18fx (12.8 billion cfu), and *Saccharomyces boulardii* 16mxg (3 billion cfu)) in combination with the addition of the enzyme alpha amylase, derived from *Aspergillus oryzae* (500 SKB (Alpha-amylase-Dextrinizing Units) in a capsule containing other inert ingredients (Rice maltodextrin, Vegetable cellulose (capsule) and Magnesium stearate), referred to hereafter as probiotic-amylase blend) led to significant (*p* ≤ 0.05) reductions in flatulence, bloating, abdominal discomfort, stool irregularity, constipation, and total severity and frequency of overall GI symptom score (GSRS) for individuals consuming a probiotic and amylase blend compared to consumption of the placebo. In addition, consumption of this blend led to a reduction in anxiety as measured by GAD-7 [7]. We previously demonstrated that filtrates from this probiotic mixture inhibited *C. albicans* germination and prevented polymicrobial biofilm formation [8]. 

The current study was designed to assess whether consumption of the novel probiotic-amylase blend during the clinical study modulated the gut microbiota composition. Thus, we obtained two stool samples: one taken at baseline prior to consuming the probiotic amylase blend, and one upon completion of a 6-week course of supplementation. We then analyzed the 16S ribosomal RNA (rRNA) gene sequences for bacterial identification, as well as the Internal Transcribed Spacer (ITS) rRNA gene sequence for identification of fungal organisms. A comparison of the gut microbiota composition pre- and post-consumption of the probiotic amylase blend is presented in the current report. Briefly, our analysis showed that participants who reported a significant improvement in clinical GI symptoms in the clinical study [7] also had a significant (*p* ≤ 0.05) fold change of *Saccharomyces cerevisiae*, a known marker of a healthy gut microbiota [200-fold and its prevalence increased (~20% to ~60%)] in members of the cohort consuming the probiotic blend. Additionally, two pathogenic bacteria, *Bacillus thuringiensis* and *Macrococcus caseolyticus*, decreased more than 150- and 175-fold, respectively, after treatment [9,10]. 

## 2. Materials and Methods

### 2.1. Experimental Design 

Fecal samples from participants of a randomized, placebo-controlled, double-blind study consisting of three study visits were obtained. The probiotic amylase blend was consumed for 6 weeks. The clinical study was conducted following ICH-GCP guidelines to ensure subject safety and scientific integrity of the data and was approved by Pearl IRB on 18 June 2021 (#21-CAHS-102); clinicaltrials.gov #NCT05614726. 

### 2.2. Study Participants 

Fifty-two (14 men and 38 women) participants were randomized into a clinical study, whose clinical outcome results have been previously reported [7]. Individuals (*n* = 27) were assigned to receive either placebo (44.7 ± 7.9 years, 169.2 ± 12.7 cm, 78.7 ± 18.0 kg, 27.4 ± 4.5 kg/m^2^) or probiotic blend (*n* = 25) (44.0 ± 10.0 years, 171.4 ± 10.3 cm, 85.5 ± 18.6 kg, 28.9 ± 4.6 kg/m^2^). All participants provided IRB-approved informed written consent prior to participating in the study. A review of health/medical history documents and a physical exam showed that all study participants were free of chronic health issues. The clinical outcomes of this study and all clinical trial-related documentation (inclusion/exclusion, subject demographics, duration of treatment, etc.) were previously reported (LaMonica et al.) [7]. 

### 2.3. Stool Kits 

At-home stool sample kits with detailed instructions were provided to all participants at their baseline visit. Participants were asked to avoid caffeine 24 h prior to collection and to collect and send their baseline stool kit prior to starting the 6-week supplementation period and at the end of the supplementation period. 

### 2.4. Microbiota Analyses

#### 2.4.1. DNA Extraction and PCR Amplification

DNA extraction and PCR amplification were performed as previously described [11,12]. Briefly, DNA was extracted using a QIA amp Fast DNA Stool Mini kit (Qiagen GmpH, Hilden, Germany) from fecal sample swabs according to the manufacturer’s instructions. The quality and purity of the isolated genomic DNA was confirmed by gel electrophoresis and quantified using a Qubit 2.0 instrument and the Qubit dsDNA HS Assay (Life Technologies, Carlsbad, CA, USA). Purified DNA samples were stored at −20 °C until used.

Amplification of the 16S and 5.8S rRNA genes was performed using primers specific for bacterial 16S-515 (5′-GGA CTA CCA GGG TAT CTA ATC CTG)-3′) and 16S -804 (5′-(TCC TAC GGG AGG CAG CAG T)-3′) as well as fungal ITS1 (5′-(TCC GTA GGT GAA CCT GCG G)-3′) and ITS4 (5′-TCC TCC GCT TAT TGA TAT GC)-3′), respectively. 

#### 2.4.2. Library Preparation and Sequencing

The amplicon library was cleaned and barcoded, followed by emulsion PCR using an Ion Torrent S5 Prime workflow according to the manufacturer’s instructions (ThermoFisher, Waltham, MA, USA), as previously reported [11,12]. Library sequencing was performed using an Ion Torrent S5 sequencer (ThermoFisher, Waltham, MA, USA).

### 2.5. Bioinformatics

#### 2.5.1. Pre-Processing

De-multiplexing of the sequencing output was performed using Python 2.7 with the input equal to the adapter-trimmed output from the Ion Torrent sequencing platform, as previously described [12]. De-multiplexed data were parsed for quality (Q20) and sequence lengths sequestered to 200–400/400–800 bp for 16S/ITS, respectively. Finally, the sequences were de-noised and the chimeras removed. Operational taxonomic units (OTUs) were generated using de novo clustering by a defined similarity threshold of 0.97. Sequences that were similar, at, or above the accepted threshold level represent the presence of a taxonomic unit (e.g., a species similarity threshold was set at 0.97) in the sequence collection.

Taxonomy was assigned using our custom pipeline based on the Greengenes V13_8 and UNITE database V7.2 taxonomic classification of 16S and ITS sequences, respectively. Blastn was used for alignment, with an error threshold of 0.001 for selection. Data were summarized, and prepared reads were represented by absolute count per ID. All analyses were performed using R Statistical Software (v4.1.2; R Core Team 2021) [13].

#### 2.5.2. Data Preparation

Raw 16S and ITS absolute count matrices were obtained from the pre-processing step, and sample annotations were loaded to R version 4.0.3. Using R package microbiome 1.12.0 and phyloseq 1.34.0 [14], a phyloseq object containing a read count matrix (species level identification OTUs), taxonomic table (kingdom down to species), and sample annotation was constructed. Initially, the 16S and ITS were handled separately until after the read counts were normalized and transformed to relative abundance.

For gut microbiota composition, stool samples at baseline and after the intervention were collected from 24 subjects assigned to probiotic-amylase blend (PRO) supplementation and 27 subjects assigned to supplementation with the placebo (PLA), consisting of rice oligodextrin (PLA). Following internal read count quality control (QC), 18 (Pre) and 21 (Post) samples from PRO and 23 (Pre) and 21 (Post) samples from the placebo (PLA) qualified for total gut microbiota comparisons of 16S (for the bacterial community) and ITS (for the fungal community). We analyzed a subset of the individuals supplementing with either the probiotic blend or placebo (*n* = 17 and 19, respectively) who provided matched Pre and Post samples that passed internal QC on total read counts. 

#### 2.5.3. Data Cleaning and QC

OTU identifiers were used to construct the taxonomic table. The table was cleaned by removing OTUs that were annotated as “unknown/unidentified” at a phyla, genus, or species level. The quality of the samples was assessed using the total read count after the taxonomic table had been cleaned and aggregated to a species level. A minimum of 500 read counts was used as the lower cut-off. Finally, samples with a read count <500 were removed, and the data were normalized and transformed to relative abundance as previously described [12].

#### 2.5.4. Data Analysis

Alpha diversity measures were calculated using the “alpha()” function, with “Shannon index” as the diversity measure, from R package microbiome, version 1.12.0. [15]. The Wilcoxon-rank-sum test “wilcox.test()” with the option “paired = TRUE” from R package stats, version 4.0.3 was used for comparisons from the same treatment group (PRO/PLA). For between-group comparisons, we used “wilcox.test()” with the option “paired = FALSE”. Fold-change between groups was calculated using the mean relative abundance of the taxa using the “foldchange()” function from R package gtools, version 3.9.2. All figures related to these data were generated using ggplot2 and ggpubr R packages, versions 3.3.5 and 0.4.0, respectively [16].

## 3. Results

### Microbiota Analyses

**Figure 1:**
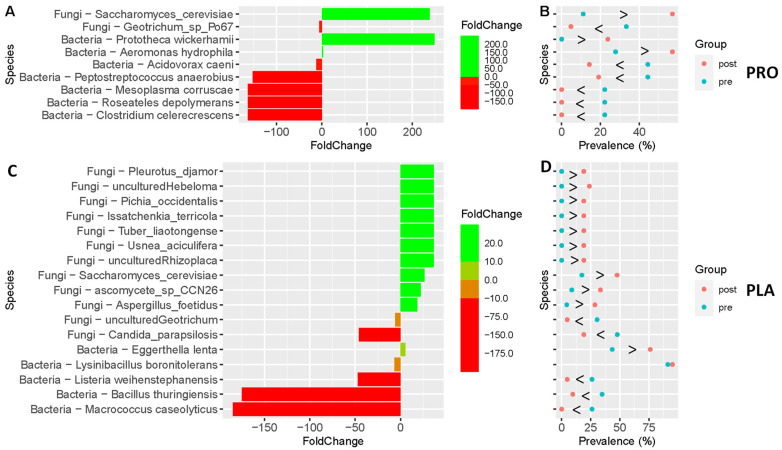
Unpaired abundance analysis of bacteria and fungi that were significantly altered (Pre versus Post) in the PRO and PLA treatment cohorts. (**A**,**B**) Taxa shown are significantly different between the Pre and Post samples of the PRO cohort (*p* < 0.05). There is a significant rise in the abundance (200-fold) of *Saccharomyces cerevisiae* (**A**), as well as an increase in its prevalence (**B**) amongst members of the PRO cohort (~20% to ~60%). (**C**,**D**) Most changes in abundance were less than 50-fold in Pre and Post timepoints of the PLA-treated cohort, with some organisms increasing while others decreased. Among fungal organisms, there was a modest increase in *S. cerevisiae* in the PLA group compared to the PRO-treated cohort and a 50-fold decrease in *Candida parapsilosis*.

Using unpaired abundance analysis, we found a number of organisms (bacteria + fungi) that were significantly altered (Pre versus Post) in individuals consuming the probiotic-amylase blend (PRO) versus those supplementing with the placebo (PLA) (Figure 1). The taxa shown in Figure 1A,B are significantly different between the Pre and Post samples in those subjects receiving the probiotic blend (*p* < 0.05). Notably, there is a significant rise in the abundance (200-fold) of *S. cerevisiae* (Figure 1A) and an increase in its prevalence (Figure 1B) amongst participants receiving the probiotic blend (~20% to ~60%). Amongst the placebo group (Figure 1C,D), most changes in abundance were less than 50-fold in Pre and Post timepoints, with some organisms increasing, while others decreased. Among fungal organisms, *S. cerevisiae* had a modest increase in individuals receiving the placebo compared to the cohort that supplemented with the probiotic blend as well as a 50-fold decrease in *Candida parapsilosis* (a known skin colonizer [17]). 

When comparing individuals who supplemented with the probiotic-amylase blend versus the placebo at 6 weeks (Post), a significantly different abundance of bacterial and fungal species was found between the two groups (*p* < 0.05). However, many of the fungi identified were listed as uncultured and may be due to poor taxonomic identification potential [18] or potentially transient detection based on the observation of Hallen-Adams and colleagues [19]. Many of the other increased fungal organisms (e.g., *Rhizoplaca*, *Aspergillus foetidus*, *Issatchenkia_terricola)* have been associated with food products or treatment [20,21,22]. 

**Figure 2:**
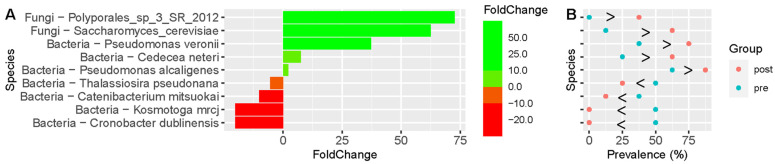
Changes in microbiota associated with clinical outcomes that were statistically different using GSRS scoring. Paired (Pre and Post) samples that passed QC for read count quality were used to examine the GSRS data. The taxa shown are significantly different between the two groups (*p* < 0.1); those organisms that achieved a *p* value of <0.05 were *S. cerevisiae*, *Cedecea neteri*, and *Pseudomonas alcaligenes*. In the GSRS response category, there was a significant increase in both (**A**) fold change (abundance) and (**B**) prevalence of the fungal organisms *S. cerevisiae* (brewer’s yeast) and Polyporales_sp_3_SR_2012, in individuals using PRO with GSRS scores greater than 1 ± standard deviation (SD) from the mean.

The taxa shown in Figure 2 are significantly different between individuals supplementing with the probiotic-amylase blend (PRO) versus those supplementing with the placebo (PLA) (*p* < 0.1); those organisms that achieved a *p* value of <0.05 were *S. cerevisiae*, *Cedecea neteri*, and *Pseudomonas alcaligenes*. Changes in these microbiota were associated with clinical outcomes that were statistically different using the Gastrointestinal Symptom Rating Scale (GSRS) scoring reported in the clinical study from which the stool samples were obtained [7]. Subjects receiving the placebo versus the probiotic amylase blend were more likely to have clinical improvement. Therefore, we examined further the clinical features assessed and performed correlation analyses with microbiota abundance.

We examined clinical features previously reported [7], including visual analog scale (VAS) variables (e.g., flatulence, bloating, stool regularity, constipation, and abdominal discomfort) to quantify individual subject microbiome outcomes within subjects receiving the probiotic amylase blend (PRO). For all clinical features in which individual subjects reported a significant improvement, a statistically significant fold change (abundance) and prevalence of *S. cerevisiae* was noted. In addition to the observed increase in *S. cerevisiae* across all clinical features, we also noted significantly different common taxa in the comparisons among clinical features, as summarized in Table 1. All individual organisms that changed significantly in Pre versus Post treatment in individuals supplementing with the probiotic amylase blend (PRO) grouped by clinical feature (flatulence, bloating, stool irregularity, constipation, and abdominal discomfort) are shown in Supplementary Figures S1–S5. 

**Figure 3:**
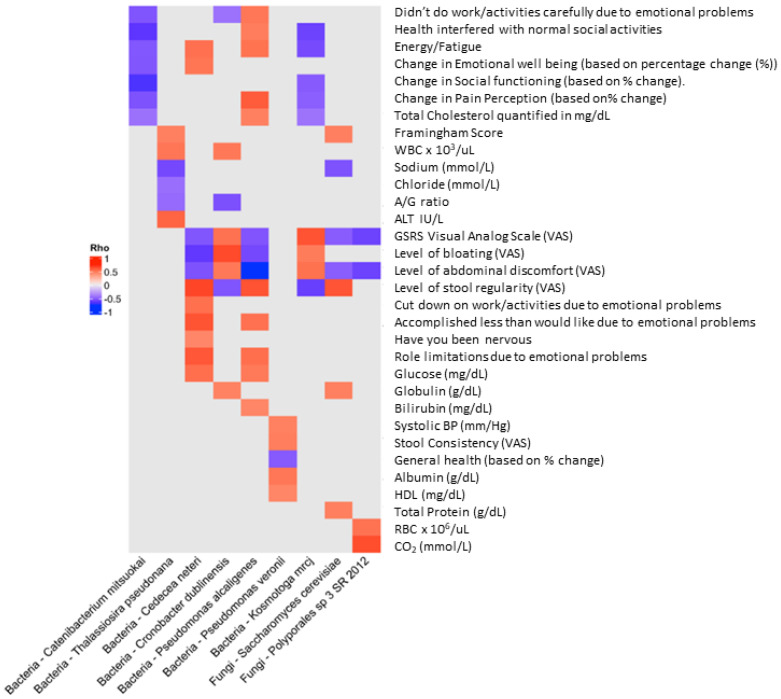
Spearman rank correlation (Rho) analysis to assess the strength of association between microbiota and clinical biomarkers. We found 9 species (7 bacterial and 2 fungal) that were significantly (negatively or positively) associated with the change among 32 clinical features. Furthermore, we note both positive and negative correlation coefficients (suggesting positive and negative relationships between the microbes and clinical variables) for the organisms identified, with the exception of *Catenibacterium mitsuokai*, which appeared to be exclusively inversely related to the clinical features assessed by VAS scores.

To determine if there were further associations between clinical features and the gut microbiota abundance, we performed Spearman rank correlation (Rho) analysis to assess the strength of association between microbiota and clinical biomarkers (Figure 3). We found nine species (seven bacterial and two fungal) that were significantly (negatively or positively) associated with the change in 32 clinical features that were originally assessed in the clinical outcomes report [7] (Figure 3). 

## 4. Discussion

This study examined the effects of a novel probiotic-amylase supplement on the modulation of the gut microbiota in a group of subjects taking the probiotic-amylase blend who exhibited mild symptoms of gastrointestinal distress (GSRS ≥ 12) [7]. In the current work we describe the effect of probiotic supplementation with the probiotic-amylase blend on the gut microbiota in this cohort. We demonstrate that consumption of the novel probiotic-amylase blend (PRO) led to positive changes in the gut microbiota, specifically leading to a highly significant increase in the abundance of *S. cerevisiae*, a known marker of a healthy gut microbiota [23,24,25], and a decrease in *Bacillus thuringiensis* and *Macrococcus caseolyticus*, as compared to supplementation with the placebo (PLA) alone.

Microbiota analyses showed a significant increase in the abundance of *S. cerevisiae* (200-fold) and an increase in its prevalence amongst those who consumed the probiotic blend as compared to those consuming the placebo (~20% at pre to ~60% at post, Figure 1A,B). Within placebo consumers, there was a slight increase in *S. cerevisiae* but a concomitant decrease in *Candida parapsilosis*, a common skin commensal organism [26]. Our observed increase in *S. cerevisiae* agrees with others who report increased abundance of *S. cerevisiae* following postbiotic supplementation and overall health improvement associated with post- or probiotic *Saccharomyces* [23,27,28]. The increase in *S. cerevisiae* abundance following probiotic-amylase blend supplementation is a robust proof of concept that oral probiotic consumption results in long-lasting changes in the microbial composition of the gut microbiota. The potential health benefits of consuming *S. cerevisiae* var. *boulardii*, especially in gut health, have been previously reported by numerous groups [11,23,29,30,31,32,33]. These benefits include modulating intestinal inflammation as well as improving host immune response. *S. cerevisiae* is a versatile fungal organism with wide-ranging scientific [34] and beneficial health implications [35,36,37].

In addition to the change in *S. cerevisiae*, we detail the changes in abundance and relationship to clinical outcome features for several organisms that were significantly increased/decreased in Pre versus Post supplementation time points following consumption of the probiotic blend. These organisms include changes in potential pathogens as well as beneficial organisms (Table 1, Supplementary Figures S1–S5). For example, *Cedecea neteri* and *Pseudomonas alcaligenes* have both been associated previously with sporadic human infections and are slightly increased following probiotic-amylase blend supplementation [38,39,40].

Furthermore, we noted both positive and negative correlation coefficients (suggesting positive and negative relationships between the microbes and clinical variables) for the organisms identified (Figure 3), with the exception of *Catenibacterium mitsuokai*, a gut commensal organism [41], which appeared to be exclusively inversely related to the clinical features assessed by visual analog scale (VAS) general well-being (e.g., energy/fatigue, emotional well-being, social functioning). This organism has also been previously associated with increased abundance associated with a high-fat (Western-style) diet [42] as well as an interesting metabolomic profile, suggesting a shared metabolic pattern with phylogenetically distant *Actinobacteria* [43]. Although most organisms were inversely related to GSRS score, bloating, and abdominal discomfort, *Cronobacter dublinensis* and *Kosmotoga mrcj*, organisms associated with emerging pathogenesis ([44,45]) and high hydrogen production and fermentation ([46,47]), respectively, were positively associated with these clinical features. It is interesting to note that *K. mrcj* was also the only organism with mixed correlations (positive and negative), where the majority of the interactions were inverse relationships to the clinical features. The organism with the highest number of correlations was *Pseudomonas alcaligenes*, which exhibited 10 positive correlations with various clinical features but also three inverse relationships (GSRS, bloating, and abdominal discomfort), respectively. This organism is a Gram-negative aerobe that belongs to the bacterial family *Pseudomonadaceae* and is a common soil and water inhabitant. It has also been reported as a rare opportunistic human pathogen [39,48]. Interestingly, members of this genus exhibit drug resistance-associated genes encoding carbapenemases [49] and have been reported to exhibit antifungal activity [50]. The organism with the least number of associations with clinical interactions was the fungus *Polyporales_sp_3_SR_2012*, which exhibited positive correlation with red blood cell counts and CO_2_ levels ([7]) but was inversely related to GSRS and abdominal discomfort scores. The classification of the *Polyporales* clade is hampered by a lack of a consensus classification within the group, and therefore exact identification and potential activity of this fungus are limited [38]. Interestingly, *S. cerevisiae*, present at higher abundance, was associated with several clinical features described previously (Framingham score, stool regularity, globulin levels, and total protein) [7], whereas it was inversely correlated with GSRS score and abdominal discomfort. This observation suggests that supplementation with the probiotic blend increased *S. cerevisiae* and was correlated with a decrease in GSRS score, suggesting an improvement in GI issues. Finally, *Pseudomonas veronii* appears to be inversely associated with general health and positively correlated with clinical features, including systolic blood pressure, stool consistency, and levels of circulating albumin and high-density lipoproteins (HDLs) [7]. This organism has a long evolutionary history [51] and has interestingly been demonstrated to be able to form biofilms in cooperation with *E. coli* and *S. cerevisiae* [52].

Modulation of the gut microbiota following probiotic consumption suggests that it may be possible to adjust probiotics blends that augment host microbial composition and may show efficacy as primary or adjuvant therapies for gastrointestinal diseases such as IBS, CD, or obesity. Indeed, modulating the microbiota via rational design of probiotics may benefit numerous clinical outcomes influenced by the gut microbiota, including potential immune modulation [53].

## 5. Limitations and Considerations

The robust increase observed in *S. cerevisiae* may be due to the probiotic-amylase blend containing *S. cerevisiae* var. *boulardii*, which shares more than 95% genome sequence homology with *S. cerevisiae* [29]; thus, rather than an organically increasing abundance of *S. cerevisiae*, we are actually just detecting the supplemented organism. However, given the changes in the other microbes concomitantly with *S. cerevisiae*, an alteration in endogenous microbiota is likely. In support of this, we observed many species of bacteria as well as other fungal species that were also altered following probiotic-amylase blend consumption. Species identified as uncultured suggest poor taxonomic identification that may be unreliable. Many of the organisms identified were also associated with food products and may suggest that rather than a gut microbiome response to the probiotic, what is actually being detected may relate to individual subject dietary habits. Finally, the addition of amylase to the blend is an important factor in how the ultimate activity of this probiotic blend may act, as observed previously in murine studies [54].

## 6. Conclusions

Our data demonstrate oral supplementation with the probiotic-amylase blend caused a marked increase in abundance of the beneficial yeast *S. cerevisiae* and concomitant modulation of gut-dwelling commensal bacterial organisms. Response to colonization of the gut by the beneficial yeast *S. cerevisiae*, the organism with the most consistent abundance profile increase, provides proof of concept that a beneficial commensal organism can re-align the gut microbiota. The significant increase in *S. cerevisiae* calls for further clinical studies examining the potential to modulate human health by augmenting beneficial commensal organisms such as *S. cerevisiae* through the use of targeted probiotics. Follow-up studies should include robust clinical trials with increased subject numbers and in vitro mechanism of action studies to explore the polymicrobial interactions between species.

## Figures and Tables

**Table 1:** Association of clinical features with significantly different common microbiota taxa occurring in the comparisons among clinical features following PRO treatment. Y indicates that this species was significantly different from the indicated clinical feature. Red arrows indicate increased abundance in taxa, blue arrows indicate decreased abundance in taxa (*p* ≤ 0.1, or *p* ≤ 0.05 ^^^).

Organism	Associated Clinical Features and Concomitant Increase (Inc) or Decrease (Dec) in Significantly Different Taxa											
	GSRS	Inc/Dec	Flatulence	Inc/Dec	Bloating	Inc/Dec	Stool Regularity	Inc/Dec	Constipation	Inc/Dec	Abdominal Discomfort	Inc/Dec
***Saccharomyces cerevisiae*** **^**	**Y**	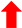	**Y**	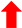	**Y**	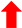	**Y**	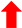	**Y**	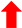	**Y**	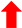
***Bifidiobacterium pseudolongum***			**Y**	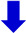	**Y**	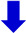	**Y**	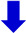	**Y**	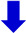	**Y**	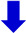
***Pseudomonas alcaligenes*** **^**	**Y**	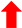			**Y**	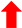	**Y**	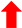	**Y**	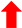		
***Clostridium aldenense*** **^**					**Y**	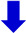	**Y**	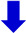	**Y**	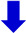	**Y**	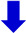
***Pseudomonas veronii***	**Y**	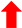					**Y**	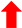	**Y**	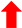		
***Cedecea neteri*** **^**	**Y**	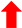					**Y**	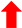			**Y**	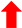
***Thalassiosira pseudonana***	**Y**	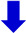	**Y**	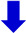	**Y**	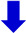						
***Paenibacillus lautus***			**Y**	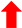	**Y**	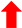					**Y**	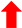
***Cryobacterium psychrophilium***			**Y**	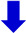	**Y**	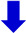					**Y**	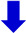
***Prevotella tannerae***			**Y**	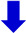	**Y**	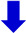					**Y**	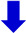
***Eubacterium biforme***					**Y**	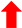	**Y**	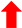	**Y**	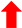		
***Rhanella aquatic*** **^**					**Y**	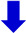	**Y**	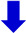	**Y**	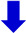		
***Bacteroides caccae*** **^**					**Y**	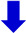			**Y**	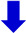	**Y**	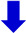
***Shigella sonnei***							**Y**	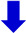	**Y**	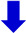		
***Pseudoalternomonas piscicida*** **^**							**Y**	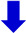	**Y**	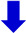		
***Serratia rubidaea***									**Y**	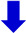	**Y**	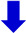

## Data Availability

All data generated or analyzed during this study are included in this published article (and its supplementary information files). The datasets generated during and/or analyzed during the current study are available from the corresponding author on reasonable request.

## Supplementary Materials

The following supporting information can be downloaded at: https://www.mdpi.com/article/10.3390/life14070824/s1, Figure S1. Statistically significant Gut microbiota changes (*p* ≤ 0.05) in Abundance (A) and Prevalence (B) for the Pre versus Post-exposure time point for PRO treatment associated with improvement in flatulence. Prevalence data with no directional arrow (>) indicates that Pre and Post values were indistinguishable. Figure S2. Statistically significant Gut microbiota changes (*p* ≤ 0.05) in Abundance (A) and Prevalence (B) for the Pre versus Post-exposure time point for PRO treatment associated with improvement in bloating. Prevalence data with no directional arrow (>) indicates that Pre and Post values were indistinguishable. Figure S3. Statistically significant Gut microbiota changes (*p* ≤ 0.05) in Abundance (A) and Prevalence (B) for the Pre versus Post-exposure time point for PRO treatment associated with improvement in stool regularity. Prevalence data with no directional arrow (>) indicates that Pre and Post values were indistinguishable. Figure S4. Statistically significant Gut microbiota changes (*p* ≤ 0.05) in Abundance (A) and Prevalence (B) for the Pre versus Post-exposure time point for PRO treatment associated with improvement in constipation. Prevalence data with no directional arrow (>) indicates that Pre and Post values were indistinguishable. Supplemental Figure S5. Statistically significant Gut microbiota changes (*p* ≤ 0.05) in Abundance (A) and Prevalence (B) for the Pre versus Post-exposure time point for PRO treatment associated with improvement in abdominal discomfort. Prevalence data with no directional arrow (>) indicates that Pre and Post values were indistinguishable.
