# Supplementary material for: A Probiotic Amylase Blend Positively Impacts Gut Microbiota Modulation in a Randomized, Placebo-Controlled, Double-Blind Study
Source: Life (Basel). 2024 Jun 28;14(7):824. doi: 10.3390/life14070824 (PMC11277872; doi:10.3390/life14070824)
Supplement: Supplementary file 1 [file life-14-00824-s001.zip › life-3029526-supplementary.pdf]

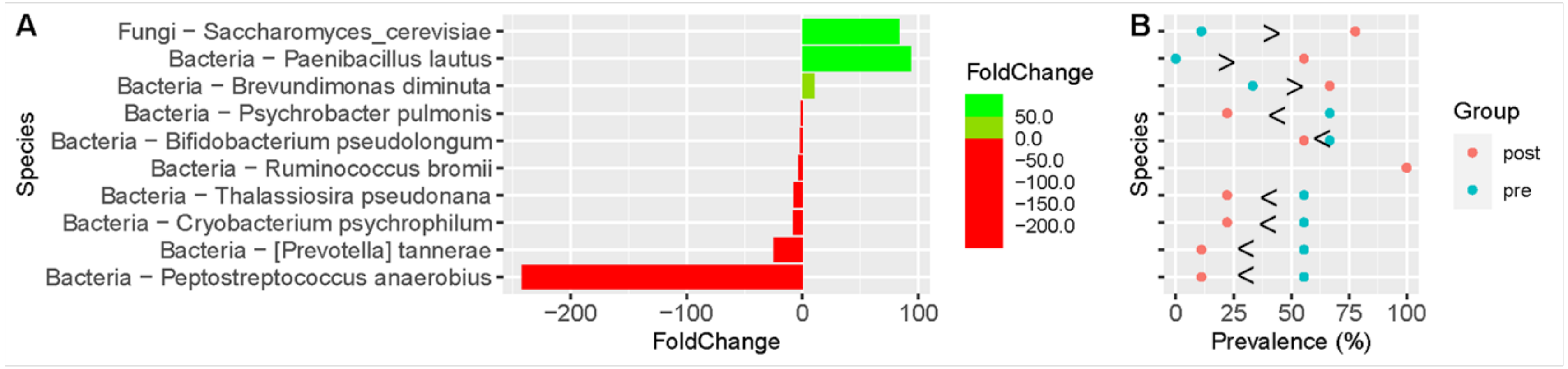

Supplemental Figure S1. Statistically significant Gut microbiota changes ($p \leq 0.05$) in Abundance (A) and Prevalence (B) for the Pre versus Post-exposure time point for PRO treatment associated with improvement in flatulence. Prevalence data with no directional arrow (>) indicates that Pre and Post values were indistinguishable.

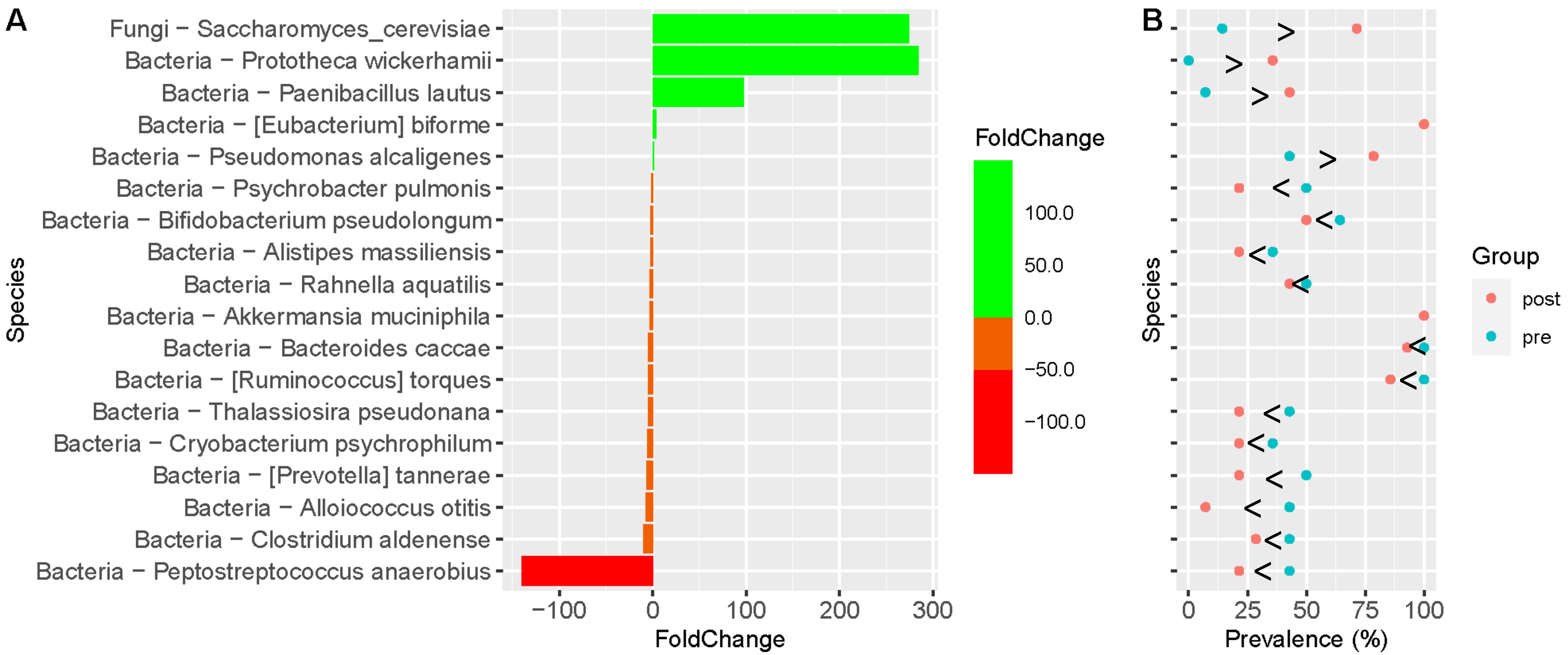

Supplemental Figure S2. Statistically significant Gut microbiota changes (p≤0.05) in Abundance (A) and Prevalence (B) for the Pre versus Post-exposure time point for PRO treatment associated with improvement in bloating. Prevalence data with no directional arrow (>) indicates that Pre and Post values were indistinguishable.

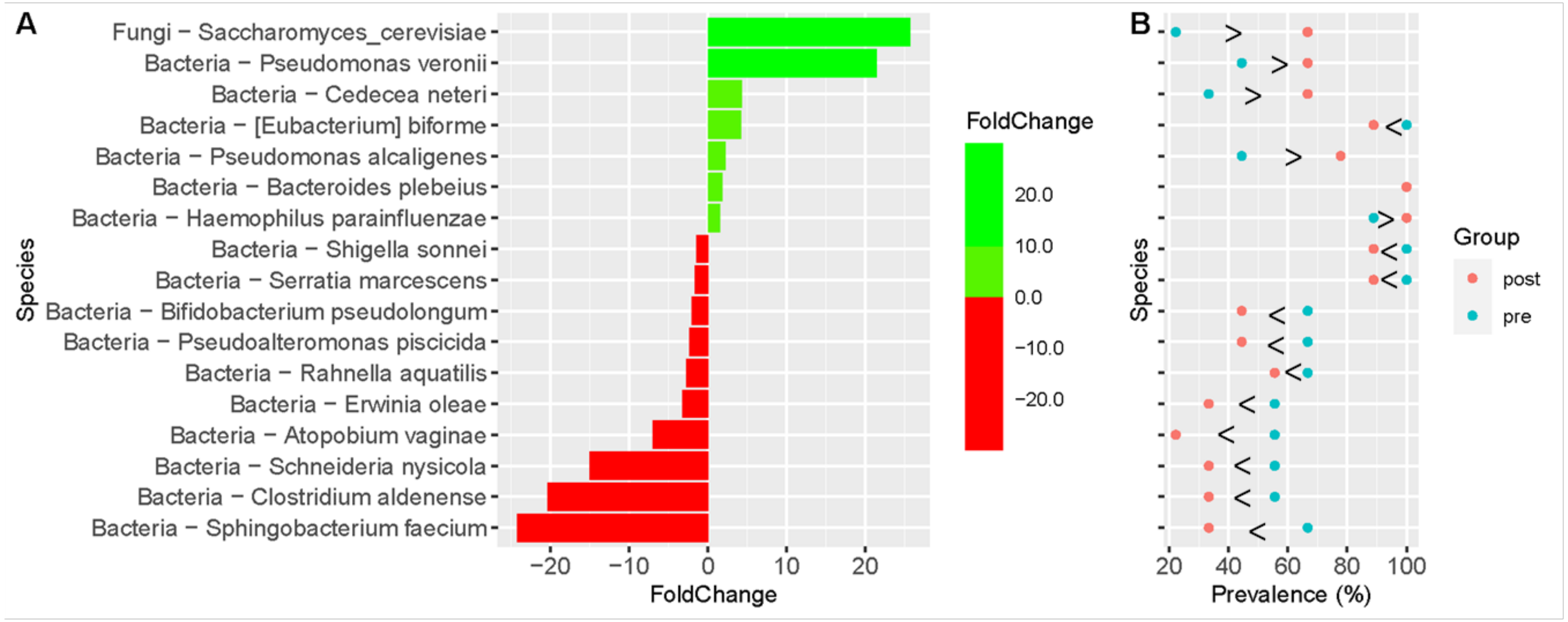

Supplemental Figure S3. Statistically significant Gut microbiota changes (p≤0.05) in Abundance (A) and Prevalence (B) for the Pre versus Post-exposure time point for PRO treatment associated with improvement in stool regularity. Prevalence data with no directional arrow (>) indicates that Pre and Post values were indistinguishable.

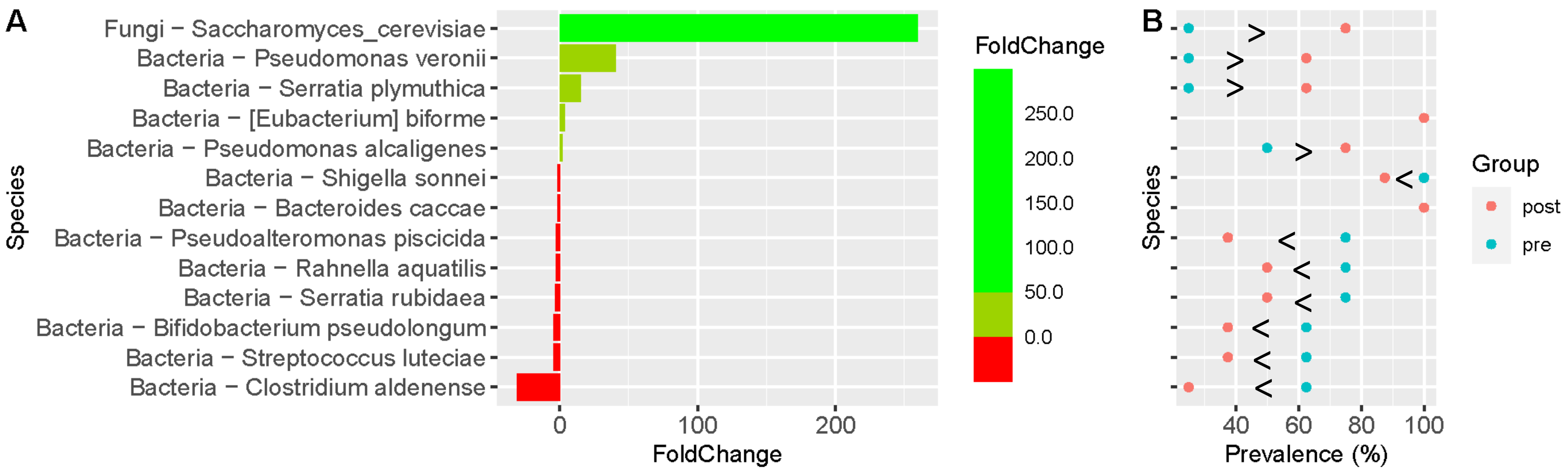

Supplemental Figure S4. Statistically significant Gut microbiota changes (p≤0.05) in Abundance (A) and Prevalence (B) for the Pre versus Post-exposure time point for PRO treatment associated with improvement in constipation. Prevalence data with no directional arrow (>) indicates that Pre and Post values were indistinguishable.

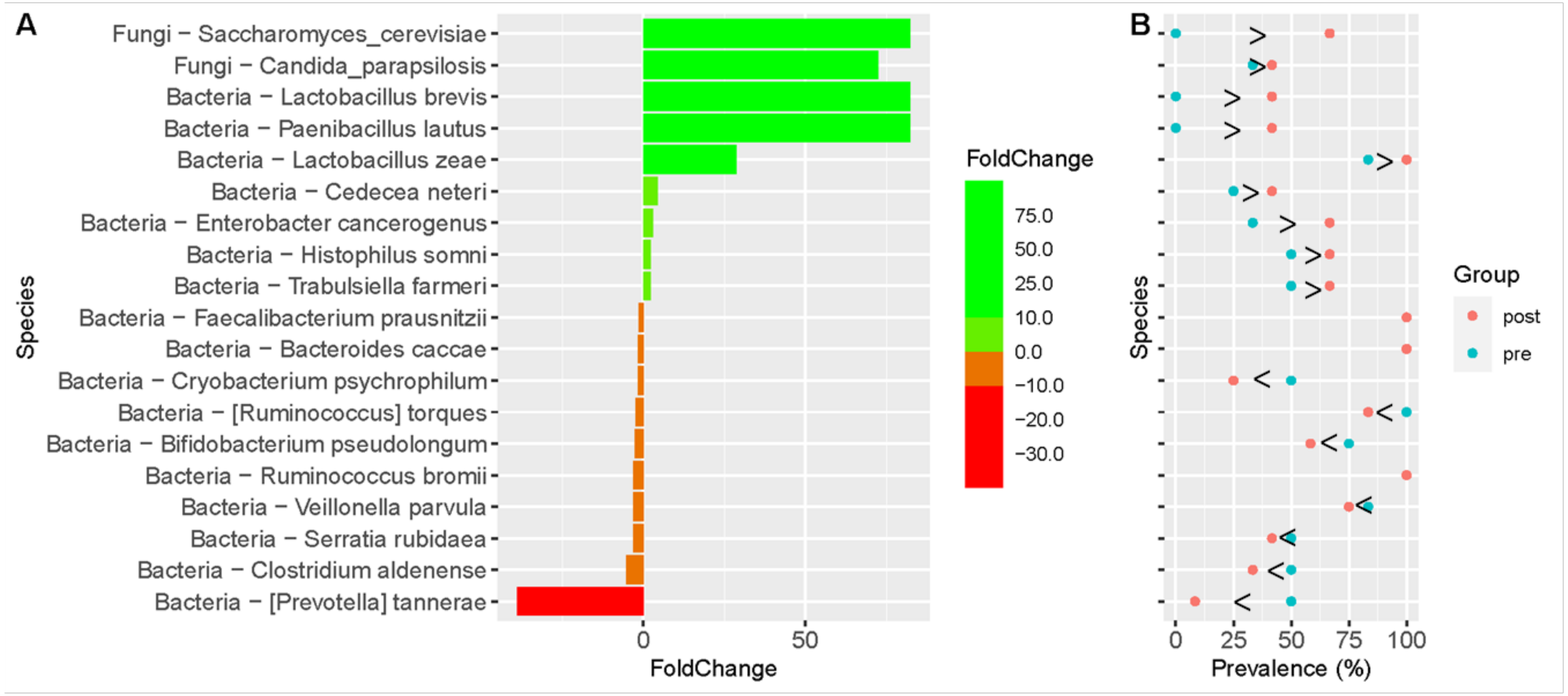

Supplemental Figure S5. Statistically significant Gut microbiota changes (p≤0.05) in Abundance (A) and Prevalence (B) for the Pre versus Post-exposure time point for PRO treatment associated with improvement in abdominal discomfort. Prevalence data with no directional arrow (>) indicates that Pre and Post values were indistinguishable.
